# Does a Gluten-Free Diet Affect BMI and Glycosylated Hemoglobin in Children and Adolescents with Type 1 Diabetes and Asymptomatic Celiac Disease? A Meta-Analysis and Systematic Review

**DOI:** 10.3390/children9081247

**Published:** 2022-08-18

**Authors:** Salma Burayzat, Nour Elsahoryi, Ali Freitekh, Osama Alzoubi, Rahaf Al-Najjar, Reema Tayyem

**Affiliations:** 1 Pediatric Gastroenterology, Hepatology and Nutrition, Faculty of Medicine, Pediatrics Department, The Hashemite University, Zarqa 13133, Jordan; 2 Faculty of Pharmacy and Medical Sciences, Nutrition Department, University of Petra, Amman 961343, Jordan; 3 Faculty of Medicine, The Hashemite University, Zarqa 13133, Jordan; 4 Faculty of Medicine, Jordan University, Amman 11942, Jordan; 5 Department of Human Nutrition, College of Health Sciences, Qatar University, Doha P.O. Box 2713, Qatar; 6 Faculty of Agriculture, University of Jordan, Amman 11942, Jordan

**Keywords:** type 1 diabetes mellitus, celiac disease, gluten-free diet, body mass index, HA1C

## Abstract

**Background:** Children diagnosed with type 1 diabetes mellitus (T1DM) are more prone to having celiac disease (CD) than the normal population. Moreover, patients with this dual diagnosis who are also on a diabetic and gluten-free diet (GFD) risk faltering growth and uncontrolled blood glucose levels. This review aims to assess the efficacy and effectiveness of managing patients with T1DM screened for CD with GFD to prevent complications associated with these chronic pathologies in childhood and adulthood. **Materials and Methods**: We abided by the PRISMA guidelines in this meta-analysis and used multiple databases and search engines. We included case–control studies. The primary outcomes were changes in the standard deviation score, body mass index (SDS BMI), and glycosylated hemoglobin (HA1C) after being on a GFD for at least twelve months. **Results:** The pooled data from the six studies included showed that there was neither a statistically significant difference in the mean SDS BMI (−0.28 (95% CI −0.75, 0.42)) (*p* = 0.24) nor in the mean of HA1C (mean −0.07 (95% CI −0.44, 0.30)) (*p* = 0.36) for the same group. HDL cholesterol improved significantly in patients on a strict GFD (*p* < 0.01). **Conclusions:** In children with T1DM and asymptomatic CD, being on a GFD had no significant effect on BMI or HA1C. However, it can have a protective effect on the other complications found in both chronic pathologies.

## 1. Introduction

The European Society for Pediatric Gastroenterology, Hepatology and Nutrition (ESPGHAN) [1] defines celiac disease (CD) as a chronic immune-mediated systemic disorder driven by gluten consumption from wheat, rye, and barley and their derivatives, which might also contaminate other grains, in the presence of specific genetic susceptibility. It presents a variable combination of clinical symptoms, specific serological markers, an HLA-DQ2/DQ8 haplotype, and enteropathy. Life-long strict adherence to a gluten-free diet leads to the disappearance of symptoms, negative titers of autoantibodies, histological recovery of the intestinal mucosa, and the elimination of long-term sequelae [2].

CD is diagnosed in pediatric patients diagnosed with type 1 diabetes mellitus (T1DM) more frequently than in the general population. In T1DM patients, CD prevalence can be up to twenty times higher than in the general population. The prevalence of CD in patients with T1DM is 3–12% [3,4].

Despite this increased risk, many health care providers struggle to reach an optimal approach for managing CD in T1DM to prevent the short- and long-term complications of these two life-long pathologies, especially in the absence of a consensus to guide their management suggestions [1]. 

Diagnosing patients with symptomatic CD, including malabsorption, and patients with subclinical disease, detected via osteopenia, growth failure, hepatic dysfunction, menstrual irregularity, unexplained epilepsy, or ataxia, remains uncomplicated. A gluten-free diet (GFD) in these symptomatic children improves their presenting signs and symptoms, making this regime strongly recommended. 

Serological autoimmune markers such as the IgA anti-tissue transglutaminase (TTG) and IgA anti-endomysial (EMA) antibodies are highly sensitive and specific, and they are now used for routine CD screening to identify ‘silent’ and ‘atypical’ forms of CD. Many of the patients identified by screening are usually asymptomatic. Evidence is inconclusive concerning the advantages versus the disadvantages of screening and treating asymptomatic individuals who are children that are already burdened with an established chronic illness [5,6].

As the development of T1DM usually precedes symptomatic and asymptomatic CD, systematic CD screening should be performed periodically in all children with T1DM [3].

GFD has been proven to benefit growth and nutrient absorption in patients with CD. On the other hand, it is unclear whether the institution of GFD in T1DM and subclinical CD patients is beneficial. Despite many screening studies on this subject, only a few have tackled the effects of putting T1DM and subclinical CD patients on a GFD. Only a handful of small prospective and retrospective studies have addressed the glycemic benefits of a GFD. Given the limited information on the role of GFD in subclinical CD cases, we decided to perform a meta-analysis and systematic review of observational case–control studies conducted on children with T1DM and asymptomatic biopsy-proven CD [7]. Children were asked to follow a GFD for at least twelve months. The primary objectives were to document the pooled effects, regardless of whether they were positive or negative, of a GFD on anthropometric parameters, mainly body mass index (BMI) and glycosylated hemoglobin (HbA1c) levels, for this group of patients. Secondary objectives were to report any other effects of a GFD in these patients, such as albumin excretion in urine, insulin dose requirement, and bone mineral homeostasis. 

To date, no review on the benefits of a GFD in children with a dual diagnosis of T1DM and asymptomatic CD has been reported before this one. We used Grading of Recommendations Assessment, Development, and Evaluation (GRADE) methods to assess the overall quality of evidence, and this method was not used in past reviews, Table A1. This systematic review and meta-analysis were registered in PROSPERO.

## 2. Materials and Methods

The studies included in this review were prospective and retrospective case–control studies; randomized control studies could not be found, as confirmed cases of CD should always be managed by a GFD and cannot be randomized to placebo management. This meta-analysis is reported according to the Preferred Reporting Items for the Systematic Reviews and Meta-Analyses (PRISMA) [8] statement and checklist (Appendix A).

The study protocol was pre-specified and registered in PROSPERO (ID: CRD42020186088) and submitted on the 5 July 2020. The registration record was published exactly as submitted. The PROSPERO team did not check for eligibility based on an exclusive focus on COVID-19 registrations during the 2020 pandemic.

We applied population, intervention, comparison, and outcome (PICO) characterization to operationalize the research questions and to determine the eligibility criteria. The inclusion criteria were as follows: children aged between one and eighteen years, an established diagnosis of T1DM at least one year before the diagnosis of CD, CD diagnosed and proven by specific antibodies and small intestinal biopsy, and prescription of a GFD for at least one year before the last assessment and follow up. As the control group of each study did not have CD, they were not put on a GFD. Studies with multiple outcomes were included. In our study, two different primary results obtained before and after adherence to a GFD for at least twelve months were studied in this review: body mass index SDS (BMI SDS) [9] was studied as an anthropometric index, and glycosylated hemoglobin (HA1C) was studied as a metabolic index. BMI SDS was used as an anthropometric index, as growth influences body weight and adiposity measurements during childhood; it is more easily standardized into SD scores (SDS) with respect to reference populations. Standard glycemic parameters such as blood glucose and glycosylated hemoglobin are used to monitor control in diabetic patients. New methods, such as glycemic variability, are emerging to aid in better management and monitoring. Most of the research in this domain still uses glycosylated hemoglobin as a metabolic parameter to follow in diabetics [10].

This review’s secondary outcomes were renal function, lipid profile, bone density, and hypoglycemic events.

The authors confirmed adherence to the GFD in subjects in the selected studies for the meta-analysis by considering the lack of symptoms in the studied subgroups. T1DM was diagnosed, and insulin therapy was initiated at least one year before confirming CD diagnosis to eliminate the effects of insulin therapy initiation on the weight and BMI of the patients. These two inclusion criteria were limiting factors in finding eligible studies.

Studies were identified using electronic sources. The following databases were used for the electronic searches: PubMed, EBSCO, the Cochrane Library, the Cochrane Central Register for Controlled Trials, Google scholar, and Springer (Appendix A). The investigation was conducted between the following dates: 28 September 2019 and 31 January 2020. Literature search strategies were developed using medical subject headings (MeSH) and free text. The complete electronic search strategy for all of the databases is presented in detail in the Appendix A.

No restrictions based on outcomes or publication were applied to the searches. The only rules applied to the searches were studies on humans, language (English and French studies included), and age from one to eighteen years. 

Three authors independently screened the titles/abstracts of potential studies for inclusion of the pre-specified inclusion/exclusion criteria. Following the initial screening, the studies’ full texts were reviewed using the same inclusion/exclusion criteria as the initial screening. A third reviewer was consulted to evaluate a study’s inclusion whenever there was a conflict between the two authors.

Studies meeting the following inclusion criteria were included in the study: study population of pediatric age group, a control group with a diagnosis of T1DM, CD confirmed by small intestinal biopsy, and patients followed for at least one year.

We assessed the quality of the overall evidence using the GRADE approach. This quality assessment method considers the study type, within-study risk of bias (methodological quality), heterogeneity, directness of evidence, precision of effect estimates, and publication bias risk. We rated the quality of the body of evidence for each key outcome as ‘high, moderate, low,’ or ‘very low’, Table A1 [11].

## 3. Statistical Analysis

The mean and standard deviation scores (SDSs) of the included studies were then input into Cochrane Review Manager Software, RevMan Version 5.4 (The Cochrane Collaboration, Copenhagen, Denmark). Weighted mean differences between the T1DM with CD groups at diagnosis and follow-up after being put on a GFD were calculated for BMI and HbA1c with 95% CIs using the random effects. In the random-effects analysis, we presumed that the actual effect size varied from one study to another. The studies in our analysis represent a random sample of effect sizes that could have been observed. The summary effect estimates the mean of these effects and accounts for heterogeneity in participant populations. Tests to determine slope heterogeneity between studies were performed, and forest plots were assessed using the x2 test and the I^2^ statistic. I^2^ values below 25% were considered to have no heterogeneity, and values up to 40% were not considered to represent significant heterogeneity. Funnel plots were performed to graphically assess potential publication bias, which was statistically evaluated with Egger’s test. Moreover, a one-way t-test was used to calculate the significant difference in the standard deviation scores (SDSs) for BMI and HbA1c among both arms (CD and T1DM at diagnosis and follow-up). A *p*-value of <0.05 was considered statistically significant for testing the pooled results of the included studies and for univariate analysis. 

## 4. Results

### 4.1. Characteristics of Included Studies

**Figure 1:**
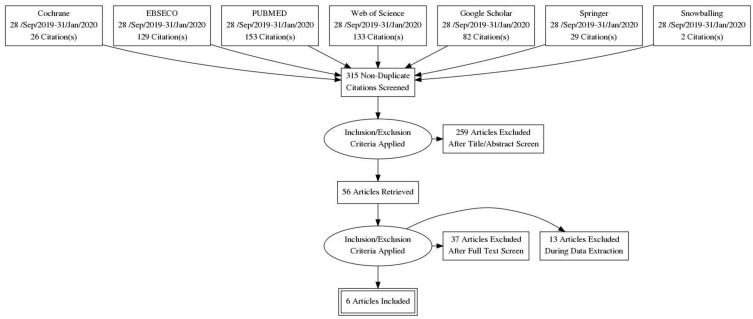
PRISMA flow diagram.

Our literature search yielded 552 titles, as shown in Figure 1. According to the PRISMA guidelines, studies were removed due to duplication, not meeting the inclusion criteria, or the statistical variables not being unified. We included six case–control studies [12,13,14,15,16,17] in the meta-analysis, as summarized in Table 1. There were five prospective [12,14,15,16,17] and one retrospective [13] case–control studies on the effects of a GFD on patients with T1DM and CD involving 578 participants. In the six studies, children who had T1DM and were found to have high titers of screening antibodies for CD and who underwent small bowel biopsies were put on a GFD and matched to children diagnosed with T1DM according to age and gender. The control groups were not put on a GFD, as the children in these groups did not have CD.

Three studies were carried out in the United Kingdom [12,13,15]. The other three were carried out in Europe [14] (in 10 pediatric diabetic centers around Europe), Australia [16], and Italy [17]. 

The primary outcome results, including the basic demographics and inclusion/exclusion criteria from the six studies, were reported in peer-reviewed journals [12,13,14,15,16,17]. The number of patients varied between studies, as shown in Table 1. The study of Rami et al. [14] had the highest number of participants (n—293 at baseline and 269 at follow-up), while the study of Amin et al. [15] had the lowest number of participants (n—33) at baseline and at follow-up. However, all of the included studies had no significant loss to follow-up, except Rami et al. [14], which had 0.1% of the participants withdraw. 

The patients’ age at CD diagnosis varied among the studies, ranging from 7.5 years in Saadah et al.’s study [16] to 13.8 years in Amin et al.’s study [15]. Regarding gender, the same percentage of females was distributed among the two groups, except in Rami et al. [14], where females accounted for 45% of the cases and 50% of the comparators. The follow-up time varied among the studies, but all of the studies had at least one year of follow-up, with Amin et al. having a longer follow-up period of 4 years [15]. According to GRADE guidelines, three studies [11,12,13] had a low risk of bias, and the other three [8,9,10] had a moderate risk of bias, Table A1. 

Concerning the age of T1DM diagnosis in the group with a dual diagnosis of T1DM and CD, only one study, Saadah et al. [16], showed an earlier T1DM diagnosis in the groups of T1DM and CD patients in comparison to the control group with T1DM. The age range at T1DM diagnosis was between four and eight years, while the range of the diagnosis age for CD was between three and seventeen years. The average duration of T1DM diagnosis before CD diagnosis was 2.3 years (1–3.1 years). 

### 4.2. Body Mass Index—SDS 

**Figure 2:**
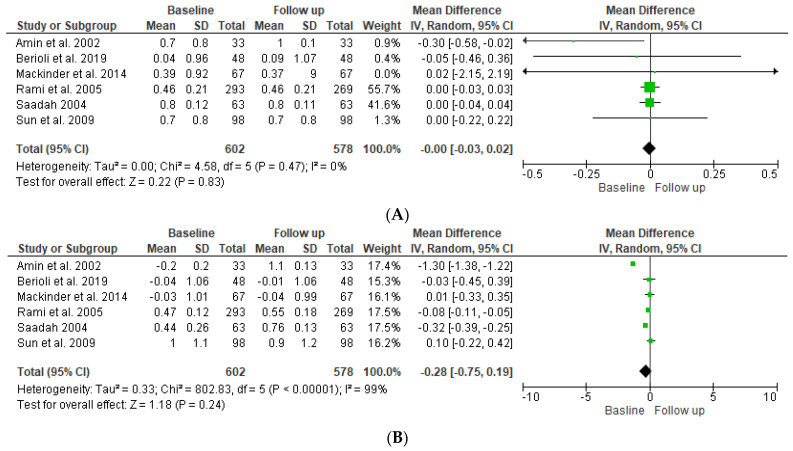
Forest plot showing standard deviation scores (SDSs) of the BMI for DM patients (**A**) and DM and CD patients (**B**) at baseline compared to follow-up [12,13,14,15,16,17].

**Figure 3:**
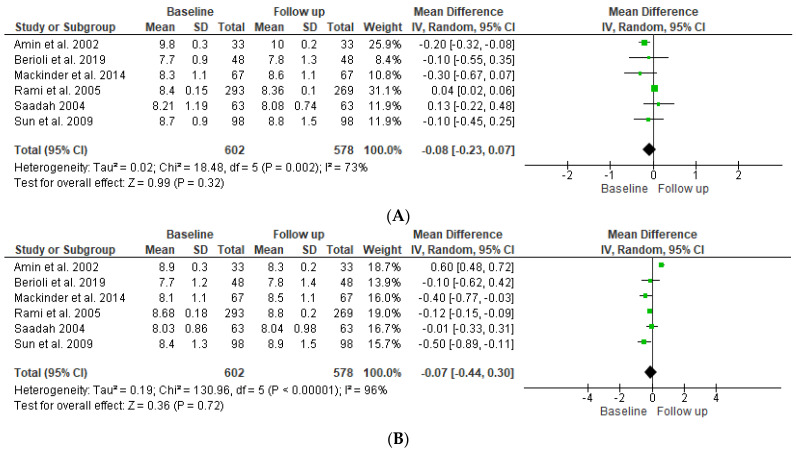
Forest plot showing HbA1c for T1DM patients (**A**) and T1DM and CD patients (**B**) at baseline compared to follow-up [12,13,14,15,16,17].

Meta-analytical findings of the first condition are described in the forest plot, as shown in Figure 2. The pooled results of the overall effect of the six studies included in this research indicated that there was no statistically significant difference in the mean (0.00 (95% CI −0.03, 0.02)) of the SDS BMI of the T1DM patients between baseline and follow-up (*p* = 0.22). The pooled results indicated no significant heterogeneity between studies (*p*-value = 0.47). The index to quantify the dispersion of the effect (I^2^) statistic = zero, reflecting minimal heterogeneity. For the T1DM and CD patients, the pooled results of the overall effect studies indicated that there was no statistically significant difference in the mean (−0.28 (95% CI −0.75, 0.42)) of the SDS BMI for the T1DM group with CD between baseline and follow-up (*p* = 0.24). The pooled results indicate that there was significant heterogeneity between studies (*p* ≤ 0.001), and the index to quantify the dispersion of the effect (I^2^) statistic = 98%, which reflects significant amounts of heterogeneity, as shown in Figure 3. 

### 4.3. Glycosylated Hemoglobin

Regarding HbA1c, as shown in the forest plot in Figure 3A, the pooled results of the overall effect of the six studies indicated that there was no statistically significant difference in the mean (mean difference −0.08 (95% −0.23, 0.07)) of the SDS BMI of T’DM patients between baseline and follow-up (*p* = 0.72). The pooled results indicate significant heterogeneity between studies (*p* ≤ 0.001), and the index to quantify the dispersion of effect (I^2^) statistic = 96%, which reflects significant heterogeneity. As shown in Figure 3B, the overall impact of both groups indicated that there was no statistically significant difference in the mean (mean difference −0.07 (95% CI −0.44, 0.30)) between baseline and follow-up ((*p* = 0.36). The pooled results indicate significant heterogeneity between studies (*p* ≤ 0.001), and the index to quantify the dispersion of effect (I^2^) statistic = 96%, which reflects significant heterogeneity. 

### 4.4. Lipid Profile, Hemoglobin Levels, Diabetic Renopathy, and Retinopathy 

As a secondary outcome, an improved lipid profile (HDL, total cholesterol, and triglyceride) after strict adherence to a GFD for at least six months was seen in three studies [18,19,20]. Hemoglobin and serum iron improved significantly after being on a GFD for at least six months [21,22]. Adherence to a GFD in patients with T1DM and CD might have a protective effect on the development of diabetic reno- and retinopathy [23,24,25,26], as seen in Table 2. 

## 5. Discussion

CD and T1DM are autoimmune pathologies where the body’s immune system produces autoantibodies that destroy its organs and tissues. These types of pathologies can affect any system in the body. Most of the time, more than one autoimmune disease can be present in the same patient concurrently, suggesting the necessity of a comprehensive management technique for multiple pathologies simultaneously. In this systematic review, a GFD did not significantly affect BMI-SDS and HA1c but showed the benefits of this specific diet on other health aspects, such as the lipid profile, diabetic retinopathy, and nephropathy.

CD and T1DM are highly associated with the HLA system, where they share common characteristics, such as the haplotypes A1, B8, DR3, and DQ2. The DQ2 locus, and mainly DQA1*0501/DQB1*, is found in over 90% of CD patients [40]. In patients with a dual diagnosis of CD and T1DM, the classic form of CD with typical gastrointestinal symptoms such as chronic diarrhea, body mass deficiency, abdominal pain, and flatulence may be observed in less than 25% of T1DM patients. However, extra-intestinal manifestations (short stature, iron deficiency anemia, or delayed puberty) and silent forms are more frequent. Recently published studies have shown that up to 71.4% of children with T1DM do not have gastrointestinal symptoms when CD-specific antibodies are detected [41,42]. International guidelines recommend CD screening at T1DM diagnosis and annually for five years [27]. In contrast, the Canadian Diabetes Association [28] recommends serologic testing based solely on clinical symptoms, including recurring gastrointestinal or extra-gastrointestinal symptoms and unexplained frequent low blood sugar levels. For adults, the recommendations are less specific [29].

In children, the most vital management technique for T1DM is dietary intervention, which can be challenging to follow and abide by in this age group [43]. In this particular group of patients, CD has a higher prevalence than in the normal population, and it is also managed by the annulment of gluten from the patient’s diet [44]. Combining these two strict dietary regimens (diabetic diet and GFD) in children can be very demanding, especially in the subgroup of patients with asymptomatic CD discovered by screening with specific antibodies and proven by small intestinal biopsy [30].

This meta-analysis and systematic review aimed to find an evidence-based approach to the effects of dietary management on these patients. The patients were diagnosed with T1DM for at least one year before CD diagnosis and starting a GFD to eliminate the effects of controlling blood sugar on weight at T1DM diagnosis that are associated with insulin therapy initiation. 

The collected data show that children with T1DM and CD lost weight after being on a gluten-free diet for at least one year. This weight loss could be explained by the difficulty of following two restrictive diets, but these studies failed to report a significant change in BMI SDS for these patients despite ensuring adherence to GFD [7,20,31]. 

Saadeh et al. illustrated that all of the anthropometric parameters of their populations were above the mean for the reference population, but none of them reached statistical significance. On the other hand, Acerini and colleagues [40] concluded that the positive benefits of dietary therapy in CD patients without gastrointestinal symptoms were uncertain after studying seven children with T1DM and CD being managed with a GFD. Westman and colleagues [45] found that dietary compliance did not change growth parameters in twenty children with T1DM and CD. Height was another anthropometric parameter that was studied. It should be noted that there were no significant changes observed in any of the studies involved in this systematic review [13,14,15,16,17,40].

This meta-analysis did not show any changes in the HA1c levels of children with a dual diagnosis of T1DM and CD after being put on a GFD. In the years before CD diagnosis, HA1c levels were usually lower than the levels measured after the confirmed diagnosis in the same patients [12]; this could be attributed to malabsorption in the absence of serum antibodies. Studies show that the presence of antibodies in the duodenal mucosa before their presence in the serum could be responsible for some of the elements of malabsorption [46,47]. However, some studies have shown an increase in HA1C levels at the beginning of GFD, but this might be explained by restoring the small intestinal mucosa and increased absorption capabilities. Nevertheless, this did not prove significant after a follow-up of at least one year [40,45,48,49].

Rami et al. [14] further subdivided the group of patients with dual diagnoses of T1DM and CD according to adherence to a GFD. Comparing the adherent and non-adherent to GFD groups, a trend toward a lower BMI SDS but not a higher z-score was noted in the non-adherent group, raising the notion of a positive influence of a GFD on weight gain in patients who adhere to a GFD.

Kaur et al. [32] reported a significant decline in the mean HbA1c level of 0.73% in the GFD group; on the other hand, it increased by 0.99% in the non-GFD group at the end of the follow-up period. Additional prospective studies have reported similar findings [15,48]. Meanwhile, other studies did not find any significant improvement in the HbA1c levels after GFD initiation [16,33]. 

Kaur et al. [32] did not report any differences in the insulin dose. At the same time, Packer et al. showed improvement in the HbA1c levels after GFD initiation compared to the controls despite no changes in the daily insulin dose being noted in their longitudinal prospective study. GFD might positively affect insulin sensitivity, explaining the improvement in HA1c values. Although the glycemic index of gluten-free and gluten-containing foods is similar [34], data suggest that the carbohydrate type might influence insulin sensitivity [50]. Moreover, for hypoglycemic events experienced before the diagnosis of CD, one study, the one by Rami et al. [14], reported no differences in the frequency of severe hypoglycemic episodes (loss of consciousness or abnormal movements) in both of the studied groups. The required insulin dosage also was not significantly altered in the group with bothT1DM and CD.

During this search, some studies did not fully meet the inclusion/exclusion criteria of this meta-analysis but still provided a growing body of evidence on the beneficial effects of a GFD for patients with concomitant T1DM and CD and indicated that it may protect against the development of other T1DM-related complications, as seen Table 2. 

Compared to T1DM patients without CD, Bakker et al. [26] showed that adult patients with TIDM and CD had a lower prevalence of retinopathy and lower total cholesterol than T1DM patients without CD. Warncke et al. [18] reported lower absolute systolic blood pressure in T1DM patients with CD than in those without CD. In the same cohort, patients with a dual diagnosis had significantly lower HDL cholesterol levels than individuals with T1DM alone at diagnosis; after the institution of a GFD, these levels increased significantly. The same effect was also noticed on bone density [51] as well as on hemoglobin and calcium levels [22]. A possible explanation for these improvements in cholesterol levels could be the normalization of the intestinal mucosa with the adoption of a GFD, demonstrating a beneficial effect of adhering to the diet.

Throughout our review, multiple papers have illustrated the possible reno-protective value of GFD on the progression of diabetic nephropathy. Malalasekera et al. [25] found that T1DM and CD patients had urinary albumin to creatinine ratios that were two-fold lower than those of patients with T1DM after adhering to a GFD for at least one year. Gopee et al. [24] reached similar findings of significantly lower urinary albumin to creatinine ratios in the same type of patients.

Although the pathophysiology of diabetic nephropathy is multifactorial, local inflammatory stress may result from both metabolic and hemodynamic derangements at a very early stage. Elmarakby et al. found significantly lower IL-1B, IL-4, and IL-5 levels in T1DM+CD patients who were adherent to a GFD than those with T1DM alone, suggesting a protective effect of a GFD on diabetic nephropathy progression [35,52]. 

Hypoglycemic episodes or a reduction in insulin requirements can be the presenting sign of CD in children with controlled T1DM. In contrast, a lack of hypoglycemic episodes at the clinical onset of CD could be the result of a higher mean blood glucose level [36]. In the six studies in this systematic review, only Rame et al. studied hypoglycemic episodes and insulin requirements. They found that no significant differences were documented after strict adherence to a GFD for at least one year of follow-up [14]. On the other hand, Mohn et al. reported a reduction in hypoglycemic events in children under a GFD [37]. 

Most of the studies in this systematic review, especially those with a large patient population, showed that a GFD normalizes the bowel mucosa and frequently leads to the disappearance of antibodies but may not necessarily lead to improved glycemic control, as seen in Table 2. 

Some studies showed a positive effect on growth parameters after adhering to a GFD for at least one year [18,19,21,38,39], yet others report no significant effect on the anthropometric indices [7,31,37,45,48,49,53]. On the other hand, long-standing CD may be associated with an increased risk of retinopathy [24], while non-adherence to a GFD may increase microalbuminuria risk [21,23,25].

A recently published study [54] reported that no significant adverse outcomes were found in children with T1DM with positive celiac serology who delayed therapy with a GFD for two years. On the other hand, the ISPAD states that in asymptomatic children with proven CD, a GFD can be considered justified to reduce the long-term risk of gastrointestinal malignancy and conditions associated with subclinical malabsorption (i.e., osteoporosis and iron deficiency) [55].

Although there were more than 500 studies found during our search due to the different search methods implemented to cover as much of the online databases as possible, only six studies met our strict inclusion criteria to decrease confounders and to have more robust results. Studies not included in online databases were not explored, as this study was not funded. Three of the six studies included in the study had low-quality evidence, and adherence to a GFD was inconsistently documented.

## 6. Conclusions

This meta-analysis and systematic review could not establish any significant positive or negative effects of a GFD on anthropometric indices or glycemic control. Meanwhile, none of the studies could individually prove a significant negative effect of a GFD on these patients. On the other hand, many clinical studies demonstrated the positive impact of a strict GFD in these patients on different health aspects, such as the possible benefit of a GFD being reno-protective and establishing a favorable atherogenic profile. Screening for CD in patients with T1DM is recommended, even in asymptomatic children. Untreated CD can lead to long-term sequelae in children with CD and in those with dual diagnoses of T1DM and CD. Further studies with large cohorts and control trials are needed to establish substantial evidence of the positive effect of GFD in children with T1DM and CD.

## Figures and Tables

**Table 1:** Main characteristics of the studies included in the meta-analysis of the effects of GFD on BMI and HA1c in children with T1DM and CD.

	Sun et al., 2009 [12]	Mackinder et al., 2014 [13]	Rami et al., 2005 [14]	Amin et al., 2002 [15]	Saadah et al., 2004 [16]	Berioli et al., 2019 [17]
Country	United Kingdom	United Kingdom	Europe	United Kingdom	Australia	Italy
Study design	Prospective case–control	Retrospective case–control	Prospective case–control	Prospective case–control	Prospective case–control	Prospective case–control
Year	2009	2014	2005	2002	2004	2019
Number of participants/controls	49/49	18/9	98/195	22/11	21/42	16/32
Recruitment of cases and controls	Pediatric diabetic units in North West England	Outpatient clinic at Royal Hospital for Sick Children Glasgow	Ten pediatric diabetic centers around Europe	Pediatric Diabetic clinic at John Radcliffe Hospital, Oxford	Royal Children’s Hospital Melbourne	Regional Centre for Children with T1DM at the Pediatric Clinic of Universita Degli Studi di, Perugia
Female (%) in the cases/comparators	63/63	52/52	45/50	54/54	62/62	56/56
The average age of cases/control (years) at the diagnosis of DM	5.9/6	5.3/5	6.5/6.5	8.1/7.4	4/NA	7.97/7.91
The average age of cases (years) at the diagnosis of CD	9.1	10.8	10	13.8	7.5	11.3
Follow up after diagnosis of CD (years)	2	2	1	4	1	1
The quality assessment GRADE system	Moderate	Moderate	Moderate	Low	Low	Low
Number of patients baseline/follow up	98/98	67/67	293/269	33/33	63/63	48/48

T1DM: type 1 diabetes mellitus; CD: celiac disease.

**Table 2:** Effects of GFD in pediatric patients with a dual diagnosis of T1DM and asymptomatic CD.

Study	[N]	Effect of GFD	*p*-Value
Warnecke et al., 2016 [16] *	974	Improvement of HDL levels Improvement in HA1C Improvement in BMI SDS.	< 0.01 <0.0001 <0.0001
Nagl et al., 2019 [17]	608	Improvement in Height SDS Improvement in Weight SDS	0.001 0.001
Salardi et al., 2017 [18]	201	Improvement in Total Cholesterol Improvement in Triglyceride	<0.025 <0.005
Hansen et al., 2006 [19]	33	Improvement in Weight SDS Mean Corpuscle Volume Hemoglobin	0.002 0.02 0.001
Gutch et al., 2016 [20]	24	Improvement in Weight SDS Improvement in Hemoglobin Improvement in serum Iron Improvement in Calcium	<0.05 <0.05 <0.05 <0.05
Acerini et al., 1998 [21] *	7	Effect on growth parameters or glycemic control	NS
Taler et al., 2012 [27]	68	No significant effect on growth and glycemic control	NS
Goh et al., 2010 [28]	29	There was no significant effect on growth parameters or glycemic control.	NS
Westman et al., 1999 [29]	20	Effect on growth parameters or glycemic control	NS
Sanchez-Albisua et al., 2005 [30]	9	Improvement in Height SDS.	0.03
Sponzilli et al., 2010 [31] *	12	Increase in Insulin requirement Increase in HA1C	0.02 <0.001
Valerio et al., 2008 [32]	57	Bone density improved significantly with strict glycemic control and adherence to GFD.	0.015
Malalasekera et al., 2009 [33]	21	Shows renoprotection of GFD	0.04
Gopee et al., 2013 [34]	24	Shows renoprotection of GFD	0.01
Mohn et al., 2001 [35]	20	Increase in Insulin requirements	0.05
Frohlich-Reiterer et al., 2011 [36]	411	Improvement in Weight SDS Improvement in Height SDS.	0.001 0.001
Narula et al., 2009 [37] *	8	Improvement in Weight-SDS Improvement in BMI-SDS	0.008 0.02
Abid et al., 2011 [38]	22	Increase in Insulin requirements	<0.005
Pham-short et al., 2014 [39]	129	Non-adherence to GFD was associated with early evidence of renal disease	0.04

N = number of patients with the dual diagnosis of T1DM and CD in the study. NS: Not significant. * Differences detected in statistics used; excluded from the forest plot.

## Data Availability

Data is available in Appendix A.

## Appendix A

*Search Strategy*

We searched Pubmed (both Medline- and Non-Medline-indexed papers), Cochrane, EBSCO, Google Scholar, Springer, and Web of Science. Two or more search queries were used in some of the databases. Our main databases were Pubmed (both Medline- and Non-Medline-indexed papers), Cochrane, EBSCO, and Web of Science. We considered Springer and Google Scholar as ‘other’ sources. We also gathered some papers through snowballing. This document is divided into three sections: Main Databases, Other Databases, and Snowballing. Each section explains the strategy used.

*Breakdown of the Strategy by Database*

1. Main Databases

A. Cochrane: *Strategy #1*

- Search Query:
  *#1 (“coeliac disease”):ti,ab,kw OR (“celiac disease”):ti,ab,kw OR (“coeliac diseases”):ti,ab,kw OR (“gluten-sensitive enteropathies”):ti,ab,kw OR (“gluten-sensitive enteropathies”):ti,ab,kw*
  *#2 (“diabetes mellitus type 1”):ti,ab,kw OR (“DM 1”):ti,ab,kw OR (“DM-1”):ti,ab,kw OR (“juvenile diabetes”):ti,ab,kw OR (“juvenile diabetes mellitus”):ti,ab,kw*
  *#3 MeSH descriptor: [Celiac Disease] explode all trees*
  *#4 MeSH descriptor: [Diabetes Mellitus, Type 1] explode all trees*
  *#5 (“gluten free diet”):ti,ab,kw OR (“gluten free diets”):ti,ab,kw OR (“gluten-free”):ti,ab,kw OR (“gluten free”):ti,ab,kw*
  *#6 MeSH descriptor: [Diet, Gluten-Free] explode all trees*
  *#7 (#4 OR #2) AND (#6 OR #5)*
  *#8 ((#4 OR #2) AND (#1 OR #3)) AND (#5 OR #6)*

- Time of Search:
  *September 28, 2019, 12:54:31.*

- Results:

9.

BMC Gastroenterology (2015) 15:181
BMJ Open 2015;5:e008097
ClinicalTrials.gov Identifier: NCT02605564
ClinicalTrials.gov Identifier: NCT02867436
Diabetes Care. 1999 Oct;22(10):1747-8
ClinicalTrials.gov Identifier: NCT03037190
Diabetes Care 25:1111–1116, 2002
ClinicalTrials.gov Identifier: NCT02605148
ClinicalTrials.gov Identifier: NCT02680054

*Strategy #2*

- Search Query:
  *#1 (“coeliac disease”):ti,ab,kw OR (“celiac disease”):ti,ab,kw OR (“coeliac diseases”):ti,ab,kw OR (“gluten-sensitive enteropathies”):ti,ab,kw OR (“gluten-sensitive enteropathies”):ti,ab,kw*
  *#2 (“diabetes mellitus type 1”):ti,ab,kw OR (“DM 1”):ti,ab,kw OR (“DM-1”):ti,ab,kw OR (“juvenile diabetes”):ti,ab,kw OR (“juvenile diabetes mellitus”):ti,ab,kw*
  *#3 MeSH descriptor: [Celiac Disease] explode all trees*
  *#4 MeSH descriptor: [Diabetes Mellitus, Type 1] explode all trees*
  *#5 (“gluten free diet”):ti,ab,kw OR (“gluten free diets”):ti,ab,kw OR (“gluten-free”):ti,ab,kw OR (“gluten free”):ti,ab,kw*
  *#6 MeSH descriptor: [Diet, Gluten-Free] explode all trees*
  *#7 (#4 OR #2) AND (#6 OR #5)*
  *#8 ((#4 OR #2) AND (#1 OR #3)) AND (#5 OR #6)*

- Time of Search:
  *September 28, 2019, 12:54:31.*

- Results:

5.

BMC Gastroenterology (2015) 15:181
BMJ Open 2015;5:e008097
ClinicalTrials.gov Identifier: NCT03037190
Diabetes Care 1999 Oct;22(10):1747-8
Diabetes Care 25:1111–1116, 2002

*Strategy #3*

- Search Query:
  *#1 MeSH descriptor: [Celiac Disease] explode all trees 338*
  *#2 MeSH descriptor: [Diabetes Mellitus, Type 1] explode all trees 5369*
  *#3 MeSH descriptor: [Diet, Gluten-Free] explode all trees 84*
  *#4 MeSH descriptor: [Glycated Hemoglobin A] explode all trees 5489*
  *#5 MeSH descriptor: [Bone Density] explode all trees 4536*
  *#6 MeSH descriptor: [Quality of Life] explode all trees 22705*
  *#7 MeSH descriptor: [Growth] explode all trees 19573*
  *#8 MeSH descriptor: [Anemia, Iron-Deficiency] explode all trees 1269*
  *#9 (Celiac):ti,ab,kw OR (Coeliac):ti,ab,kw OR (Gluten sensitive Enteropathy):ti,ab,kw 1261*
  *#10 (T1DM):ti,ab,kw OR (Type 1 DM):ti,ab,kw OR (Type 1 Diabetes Mellitus):ti,ab,kw OR (Juvenile Diabetes):ti,ab,kw OR (Insulin Dependent Diabetes):ti,ab,kw 38096*
  *#11 (GFD):ti,ab,kw OR (Gluten Free):ti,ab,kw 550*
  *#12 (Glycemic control):ti,ab,kw OR (HbA1C):ti,ab,kw 25407*
  *#13 (BMD):ti,ab,kw OR (Bone Mineral Density):ti,ab,kw OR (Bone Density):ti,ab,kw 12701*
  *#14 (QOL):ti,ab,kw OR (Quality of Life):ti,ab,kw 112273*
  *#15 (Growth):ti,ab,kw 45637*
  *#16 (Iron Deficiency Anemia):ti,ab,kw 2988*
  *#17 (#1 OR #9) AND (#2 OR #10) AND (#3 OR #11) AND (#4 OR #5 OR #6 OR #7 OR #8 OR #12 OR #13 OR #14 OR #15 OR #16) 12*

- Time of Search:
  *February 7, 2020, 15:33:48.*

- Results:

12.

BMJ open, 2015, 5(5), e008097
BMC gastroenterology, 2015, 15, 181
BMJ open, 2015, 5(5), e008097
Journal of pediatric gastroenterology and nutrition, 2017, 64(2), 175-179
Pediatric diabetes 2018;19:49
https://clinicaltrials.gov/show/NCT01566110, 2012
BMC gastroenterology 2015;15(1): Article number: 181
Pediatric diabetes 2014; 15: 114
https://www.who.int/trialsearch/Trial2.aspx?TrialID=CTRI/2018/06/014508
Diabetes care, 2011, 34(6), 1301-1305
Pediatric Diabetes 2016;17(S24):33-34
Diabetes care 1999;22(10):1747-1748

B. EBSCO:

*Strategy #1*

- Search Query:
  *S1: diabetes mellitus type 1 OR dm type 1 OR juvenile diabetes OR insulin dependent diabetes OR iddm*
  *S2: celiac disease OR gluten sensitive OR gluten enteropathy OR coeliac disease*
  *S3: gluten free diet OR special diets OR gluten free OR gfd*
  *S4: glycemic control OR hba1c OR glucose*
  *tolerance test OR blood glucose levels OR quality of life*
  *S5: S1 AND S3 AND S4*
  *S6: S1 AND S2 S3 AND S4*
  *Limit for paper date: 1 January 2011–31 December 2020.
  **Databases Searched: Business Source Complete, Academic Search Ultimate, Art & Architecture Complete, MEDLINE Complete, Education Research Complete, Communication & Mass Media Complete, Environment Complete, Computers & Applied Sciences Complete, Legal Collection, Energy & Power Source, Research Starters—Education, Research Starters—Business.*

- Time of Search:
  *October 11, 2019, 5:13:41 AM.*

- Results:

81.

Food Reviews International. 2019, Vol. 35 Issue 6, p587-608
SpringerPlus (2016) 5:994
BMC Res Notes (2019) 12:50
BMC Gastroenterology (2015) 15:181
Indian J Med Res 149, January 2019, pp 18-25
BMJ Open 2015;5:e008097
BMC Gastroenterology 2014, 14:99
Diabetes Ther (2019) 10:1151–1161
British Journal of Nursing, 2019, Vol 28, No 15, 1015-1019
Diabetes Therapy. Feb2019, Vol. 10 Issue 1, p119-134
Acta Diabetol (2017) 54:889–894
PLoS **ONE**. Nov2013, Vol. 8 Issue 11, p1-9
Diabetic Medicine: A Journal Of The British Diabetic Association [Diabet Med] 2014 Feb; Vol. 31 (2), pp. 208-12
Ethiop J Health Sci. Vol. 29, No. 4, 447-452
Pediatric Diabetes [Pediatr Diabetes] 2011 Jun; Vol. 12 (4 Pt 1), pp. 322-5
Journal of Diabetes Research. 12/20/2018, p1-11
J Diabetes Investig 2019; 10: 104–107
Turk J Endocrinol Metab 2017;21:127-130
BMJ Case Reports 2012; doi:10.1136/bcr.02.2012.5878
JURNALUL PEDIATRULUI – Year XVII, Vol. XVII, Supplement 1, 2014
Pediatric Diabetes [Pediatr Diabetes] 2012 Dec; Vol. 13 (8), pp. 597-606
Acta Diabetol (2013) 50:319–324
Pediatric Diabetes [Pediatr Diabetes] 2012 Mar; Vol. 13 (2), pp. 163-9
Scientific Reports volume7, Article number: 45286 (2017)
Diabetes Obes Metab. 2019;21:1769–1779
Diabetes Care 34:2158-2163, 2011
Journal of Diabetes 9 (2017), 865–873
Diabetes Care 2015;38:801–807
Diabetes Ther (2017) 8:875–886
Diabetol Metab Syndr (2016) 8:51
International Journal of Health Sciences Vol. 11, Issue 2 (April–June 2017), 65-71
Nutrition & Metabolism (2019) 16:60
ANNALS VOL 23, ISSUE 3, JULY–SEPT. 2017, 289-293
Dig Dis Sci (2012) 57:1314–1320
Nutrition & Metabolism (2017) 14:11
JAMA August 15, 2017 Volume 318, Number 7, 637-646
Aust N Z J Obstet Gynaecol 2019; 59: 208–214
Indian Journal of Endocrinology and Metabolism (2016), 443-450
Am J Clin Nutr 2019;109:288–296
Nutrition Journal 2013, 12:29
Pediatric Diabetes [Pediatr Diabetes] 2016 May; Vol. 17 (3), pp. 191-8
Diabetic Medicine. Jul2013, Vol. 30 Issue 7, p835-839
Lipids in Health and Disease (2016) 15:78
Food & Nutrition Research 2016, 60: 32594
Diabetic Medicine. May2018, Vol. 35 Issue 5, p541-547
Nutrition Journal (2018) 17:42
Asia Pac J Clin Nutr 2018;27(3):728-734
Vojnosanit Pregl 2012; 69(10): 858–863
J Community Health (2016) 41:584–592
Diabet. Med. 36, 653–654 (2019)
Pediatric Diabetes. Sep2013, Vol. 14 Issue 6, p455-458
Diabetes Care 2016;39:808–815
J Nippon Med Sch 2013; 80 (3)
Journal of Nutrition & Metabolism. 2012, p1-21
International Journal of Endocrinology. 2013, p1-7
Eur J Nutr (2013) 52:813–824
Journal of Jahrom University of Medical Sciences. 2012, Vol. 9 Issue 4, following p7-7
Am J Clin Nutr 2017;106:1394–400
Diabetes 2017;66:1373–1379
Nutrition & Metabolism (2018) 15:80
Diabetic Medicine. Nov2017, Vol. 34 Issue 11, p1554-1567
Journal of Human Nutrition & Dietetics. Jun2017, Vol. 30 Issue 3, p385-393
Diabetic Medicine. Sep2015, Vol. 32 Issue 9, p1149-1155
Journal of Clinical Pharmacy and Therapeutics (2011) 36, 592–601
BMC Public Health (2018) 18:525
Diabetes 2017;66:627–639
British Journal of Nutrition (2018), 119, 910–917
Diabetes, Obesity & Metabolism. Jul2014, Vol. 16 Issue 7, p577-587
Journal of Human Nutrition & Dietetics. Apr2014 Supplement, p21-27
Diabetes 2017;66:75–86
Ann Nutr Metab 2011;58:74–78
British Journal of Nursing, 2017, Vol 26, No 10, 543-551
Diabetes Care 2014;37:1824–1830
Diabetes Care 2016;39:893–901
South African Journal of Clinical Nutrition. 2016, Vol. 29 Issue 2, p90-94
Australian and New Zealand Journal of Obstetrics and Gynaecology 2016; 56: 333–335
Geriatr Gerontol Int 2012; 12 (Suppl. 1): 41–49
Journal of Cystic Fibrosis 15 (2016) 261–262
ACP Journal Club. 2/17/2015, Vol. 162 Issue 4, p1-1
American Journal of Clinical Nutrition. Mar2013, Vol. 97 Issue 3, p453-454
Diabetes Health. Oct/Nov2011, Vol. 20 Issue 5, p20-20

*Strategy #2*

- Search Query:
  *S1: diabetes mellitus type 1 OR dm type 1 OR juvenile diabetes OR insulin dependent diabetes OR iddm*
  *S2: celiac disease OR gluten sensitive OR gluten enteropathy OR coeliac disease*
  *S3: gluten free diet OR special diets OR gluten free OR gfd*
  *S4: glycemic control OR hba1c OR glucose*
  *tolerance test OR blood glucose levels OR quality of life*
  *S5: S1 AND S3 AND S4*
  *S6: S1 AND S2 S3 AND S4*
  *Limit for paper date: 1 January 2011–31 December 2020
  **Databases Searched: Business Source Complete, Academic Search Ultimate, Art & Architecture Complete, MEDLINE Complete, Education Research Complete, Communication & Mass Media Complete, Environment Complete, Computers & Applied Sciences Complete, Legal Collection, Energy & Power Source, Research Starters—Education, Research Starters—Business*

- Time of Search:
  *October 11, 2019, 5:13:41 AM.*

- Results:

24.

Food Reviews International 2019, Vol. 35 Issue 6, p587-608
Indian J Med Res 149, January 2019, pp 18-25
BMJ Open 2015;5:e008097
BMC Gastroenterology (2015) 15:181
BMC Gastroenterology 2014, 14:99
Acta Diabetol (2017) 54:889–894
Diabetic Medicine: A Journal Of The British Diabetic Association [Diabet Med] 2014 Feb;Vol. 31 (2), pp. 208-12
Pediatric Diabetes [Pediatr Diabetes] 2011 Jun; Vol. 12 (4 Pt 1), pp. 322-5
Scientific Reports volume7, Article number: 45286 (2017)
Pediatric Diabetes [Pediatr Diabetes] 2012 Dec; Vol. 13 (8), pp. 597-606
Diabetes Care 34:2158-2163, 2011
Pediatric Diabetes [Pediatr Diabetes] 2012 Mar; Vol. 13 (2), pp. 163-9
Diabetes Care 2015;38:801–807
Dig Dis Sci (2012) 57:1314–1320
Pediatric Diabetes [Pediatr Diabetes] 2016 May; Vol. 17 (3), pp. 191-8
Turk J Endocrinol Metab 2017;21:127-130
Diabetol Metab Syndr (2016) 8:51
JURNALUL PEDIATRULUI – Year XVII, Vol. XVII, Supplement 1, 2014
Acta Diabetol (2013) 50:319–324
ANNALS VOL 23, ISSUE 3, JULY–SEPT. 2017
Diabetic Medicine. Jul2013, Vol. 30 Issue 7, p835-839
BMJ Case Reports 2012; doi:10.1136/bcr.02.2012.5878
Pediatric Diabetes. Sep2013, Vol. 14 Issue 6, p455-458
Diabetes Health. Oct/Nov2011, Vol. 20 Issue 5, p20-20

*Strategy #3*

- Search Query:
  *S1: Celiac OR Coeliac OR Gluten Sensitive Enteropathy*
  *S2: T1DM OR Type 1 DM OR Type 1 Diabetes Mellitus OR Juvenile Diabetes OR Insulin Dependent Diabetes*
  *S3: GFD OR Gluten Free*
  *S4: Glycemic Control OR HBA1C OR BMD OR Bone Mineral Density OR Bone Density OR QOL OR Quality of Life OR Growth OR Iron Deficiency Anemia*
  *S5: S1 AND S2 AND S3 AND S4*
  **Databases Searched: MEDLINE Complete, eBook Collection (EBSCOhost), MEDLINE, eBook Academic Collection (EBSCOhost), CINAHL Plus with Full Text*

- Time of Search:
  January 31, 2020. 3:00 PM.

- Results:

24.

Diabetic Medicine Dec2009; 26(12): 1250-1254
Journal of Pediatric Gastroenterology & Nutrition Feb2017; 64(2): 175-179
BMC Gastroenterology 12/21/2015; 15: 181
Pediatric Diabetes Dec2012; 13(8): 597-606
Diabetic Medicine Jul2013; 30(7): 835-839
Acta Paediatrica Apr 2017; 106(4): 639-646
Diabetes Care Jul2002; 25(7): 1117-1122
Pediatric Diabetes Jun2011; 12(4pt1): 322-325
Pediatric Diabetes May2016; 17(3): 191-198
Healy, Shavonne Access Nov2014; 28(9): 14-15 NLM UID: 9885733
Diabetic Medicine Aug2005; 22(8): 1079-1082
http://dx.doi.org/10.1136/bcr-2013-200472
Archives of Disease in Childhood Sep2004; 89(9): 871-876
Diabetes Care Oct2011; 34(10): 2158-2163.
Turkish Journal of Endocrinology & Metabolism 2017; 21(4): 127-130
Digestive Diseases & Sciences May2012; 57(5): 1314-1320
BMC Gastroenterology 2014; 14(1): 99 https://doi.org/10.1186/1471-230X-14-99
Practical Diabetes International Apr2011; 28(3): 110-112
http://dx.doi.org/10.1136/adc.87.6.495
Gastroenterology & Hepatology from Bed to Bench Autumn2014; 7(4): 189-197
Pediatric Diabetes Mar2012; 13(2): 163-169
BioMed Research International 2013; 2013: 127589-127589
Clinical Medicine & Research Oct2007; 5(3): 184-192
British Journal of Diabetes & Vascular Disease Mar2008; 8(2): 67-71

C. Pubmed:

*Strategy #1*

- Search Query:
  *(((“Diabetes Mellitus, Type 1”[MeSH Terms] AND (“Diet, Gluten-Free”[MeSH Terms] OR “Foods, Specialized”[MeSH Terms] OR “specialized diet”[All Fields]* *OR “special diet”[All Fields] OR “modified diet”[All Fields] OR “selective diet”[All Fields] OR “restrictive diet”[All Fields] OR “restricted diet”[All Fields] OR “restricted food intake”[All Fields] OR “restricted dietary intake”[All Fields] OR (restricted[All Fields] AND (“diet”[MeSH Terms] OR “diet”[All Fields] OR “dietary”[All Fields]) AND practices[All Fields]))) AND (“Blood Glucose”[MeSH Terms] OR “Hyperglycemia”[MeSH Terms] OR “Glucose Tolerance Test”[MeSH Terms] OR “Glycated Hemoglobin A”[MeSH Terms] OR “Diabetes Complications”[MeSH Terms] OR “Quality of Life”[MeSH Terms] OR “Life Style”[MeSH Terms] OR “Hospitalization”[MeSH Terms] OR “glycemic control”[All Fields] OR “re admission”[All Fields] OR “blood sugar control”[All Fields] OR “blood glucose control”[All Fields] OR “hospital admission”[All Fields])) AND (“humans”[MeSH Terms] AND (“infant”[MeSH Terms] OR “child”[MeSH Terms] OR “adolescent”[MeSH Terms]))) OR (((((“celiac”[All Fields] OR “coeliac”[All Fields] OR “celiac sprue”[All Fields] OR “nontropical sprue”[All Fields] OR (gluten-sensitive[All Fields] AND enteropath[All Fields]) OR “gluten-induced enteropathy”[All Fields] OR “gluten induced enteropathy”[All Fields]) OR (“type 1 diabetes mellitus”[All Fields] OR “type 1 diabetes”[All Fields] OR “juvenile diabetes”[All Fields] OR “t1dm”[All Fields] OR “t1d”[All Fields] OR “type-1 diabetes”[All Fields] OR “insulin dependent diabetes”[All Fields] OR “insulin dependant diabetes”[All Fields] OR “insulin-dependent diabetes”[All Fields] OR “insulin-dependant diabetes”[All Fields])) AND (“gluten-free diet”[All Fields] OR “gfd”[All Fields] OR “gluten free diet”[All Fields] OR “specialized diet”[All Fields] OR “restrictive diet”[All Fields] OR “restricted diet”[All Fields] OR “special diet”[All Fields] OR “gf diet”[All Fields] AND “specialized diet”[All Fields] OR “special diet”[All Fields] OR “modified diet”[All Fields] OR “selective diet”[All Fields] OR “restrictive diet”[All Fields] OR “restricted diet”[All Fields] OR “restricted food intake”[All Fields] OR “restricted dietary intake”[All Fields] OR (restricted[All Fields] AND (“diet”[MeSH Terms] OR “diet”[All Fields] OR “dietary”[All Fields]) AND practices[All Fields]))) AND (“blood glucose”[All Fields] OR “hyperglycemia”[All Fields] OR “glucose tolerance test”[All Fields] OR “glycated hemoglobin A”[All Fields] OR “diabetes complications “[All Fields] OR “quality of life”[All Fields] OR “Life Style”[All Fields] OR “hospitalization”[All Fields] OR “glycemic control”[All Fields] OR “re admission”[All Fields] OR “blood sugar control”[All Fields] OR “blood glucose control”[All Fields] OR “hospital admission”[All Fields] OR “qol”[All Fields] OR “hba1c”[All Fields] OR “blood sugar”[All Fields] OR “re admission”[All Fields] OR (hemoglobyn[All Fields] AND a1c[All Fields]))) NOT medline[sb]) AND (“humans”[MeSH Terms] AND (“infant”[MeSH Terms] OR “child”[MeSH Terms] OR “adolescent”[MeSH Terms]))*

- Time of Search:
  October 4, 2019.

- Results:

29.

Scientific Reports | 7:45286, 2017, 1-7
J Pediatr. 2016 Dec;179:131-138
Kylökäs et al. BMC Gastroenterology (2016) 16:76
Assor et al. BMC Gastroenterology (2015) 15:181, 1-10
Diabet Med. 2016 Jul;33(7):947-55
Nutr Hosp. 2015;32(2):634-637
BMJ Open 2015;5:e008097, 1-8
BMC Gastroenterology 2014, 14:99, 1-8
J Obstet Gynecol Neonatal Nurs. 2013 Nov-Dec;42(6):619-28
Diabet Med. 2014 Feb;31(2):208-12
Pediatr Diabetes 2013 Sep;14(6):455-8
BMJ Case Reports 2012, 1-3
Pediatr Diabetes 2012 Dec;13(8):597-606
Dig Dis Sci. 2012 May;57(5):1314-20
DIABETES CARE, VOLUME 34, OCTOBER 2011, 2158-2163
Pediatr Diabetes. 2012 Mar;13(2):163-9
J Pediatr Endocrinol Metab. 2010 Nov;23(11):1169-73
Dtsch Med Wochenschr. 2011 Feb;136(5):172-5
Acta Biomed. 2010 Dec;81(3):165-70
Diabet Med. 2009 Dec;26(12):1250-4
Diabetologia (2009) 52:798–800
Rev Latino-am Enfermagem 2005 janeiro-fevereiro; 14(1)
European Journal of Clinical Nutrition (2004) 58, 1429–1431
Atherosclerosis. 1999 Aug;145(2):389-97
J Am Diet Assoc. 1996 May;96(5):458-63
Nurse Pract Forum. 1991 Sep;2(3):193-5
Diabetes Care. 1987 Jan-Feb;10(1):33-8
Zentralbl Gynakol. 1986;108(11):691-8
Diabetes. 1975 Jul;24(7):672-9

*Strategy #2*

- Search Query”
  *(“Celiac Disease”[Mesh] AND “Diabetes Mellitus, Type 1”[Mesh]) AND (“Diet Therapy”[Mesh] OR “diet therapy”[Subheading]) AND (“2009/12/03”[PDat]: “2019/11/30”[PDat] AND (“infant”[MeSH Terms] OR “child”[MeSH Terms] OR “adolescent”[MeSH Terms]))*

- Time of Search:
  *November 30, 2019.*

- Results:

40.

Indian J Med Res. 2019 Jan;149(1):18-25
Pediatr Diabetes. 2019 May;20(3):293-303
Can J Diet Pract Res. 2018 Sep 1;79(3):118-124
Acta Diabetol. 2017 Oct;54(10):889-894
Sci Rep. 2017 Mar 24;7:45286
J Pediatr Gastroenterol Nutr. 2017 Aug;65(2):195-199
J Pediatr. 2016 Dec;179:131-138
BMC Gastroenterol. 2016 Jul 25;16(1):76
Rev Gaucha Enferm. 2016 Mar;37(1):e53787
J Diabetes Complications. 2016 Mar;30(2):295-9
BMC Gastroenterol. 2015 Dec 21;15:181
Lik Sprava. 2015 Jan-Mar;(1-2):167-9
BMJ Open. 2015 May 11;5(5):e008097
Diabetes Care. 2015 May;38(5):760-6
Diabetes Care. 2014 Sep;37(9):e194-5
J Paediatr Child Health. 2014 Oct;50(10):811-6
BMC Gastroenterol. 2014 May 28;14:99
Nutrients. 2013 Nov 18;5(11):4540-52
Eur J Clin Invest. 2014 Jan;44(1):74-82
Diabet Med. 2014 Feb;31(2):208-12
Indian J Gastroenterol. 2013 Sep;32(5):330-4
Pediatr Diabetes. 2013 Sep;14(6):455-8
Indian J Gastroenterol. 2014 Mar;33(2):188-9
J Gastroenterol Hepatol. 2013 Jan;28(1):99-105
Indian J Gastroenterol. 2013 Mar;32(2):98-102
Pediatr Diabetes. 2012 Dec;13(8):597-606
Clin Exp Immunol. 2012 Feb;167(2):226-34
Dig Dis Sci. 2012 May;57(5):1314-20
Diabetes Care. 2011 Oct;34(10):2158-63
Transl Res. 2011 Sep;158(3):140-5
Pediatr Diabetes. 2012 Mar;13(2):163-9
Pediatr Diabetes. 2011 Jun;12(4 Pt 1):322-5
Diabetes Care. 2011 Jun;34(6):1301-5
J Pediatr Endocrinol Metab. 2010 Nov;23(11):1169-73
J Gastroenterol Hepatol. 2011 Feb;26(2):378-81
Diabetes Res Clin Pract. 2011 Apr;92(1):53-6
Acta Biomed. 2010 Dec;81(3):165-70
J Pediatr. 2011 Feb;158(2):276-81.e1
Gastroenterol Clin Biol. 2010 Apr-May;34(4-5):319-20.
Rheumatol Int. 2010 Apr;30(6):793-5

*Strategy #3*

- Search Query
  *((((((“diabetes mellitus, type 1”[mesh]) and “celiac disease”[mesh]))) AND (((((“diet therapy”[mesh]) or “nutrition therapy”[mesh]) or “diet, gluten-free”[mesh]))))) AND (((((((readmission) OR hospital readmission*s) OR “Patient Readmission”[Mesh]) OR patient readmission)) OR ((((((blood glucose) OR “Blood Glucose”[Mesh]) OR hypoglycemic control) OR glycemic control) OR diabetes management)) OR ((((((quality of life) OR “Quality of Life”[Mesh]) OR life quality) OR health quality)))) OR ((“diabetes “ and “celiac “ and “gluten” and “diet” NOT medline[sb]))*

- Time of Search:
  *November 18, 2019.*

- Results:

*63.*

Diabetes Care. 2016 Aug;39(8):e119-20.
JAMA. 2016 Sep 20;316(11):1181-1192.
World J Diabetes. 2013 Aug 15;4(4):130-4.
doi: 10.2174/0929867326666190409120716.
Gastroenterol Hepatol Bed Bench. 2014 Fall;7(4):189-97.
World J Diabetes. 2015 Jun 10;6(5):707-14.
Horm Res Paediatr. 2019 Oct 8:1-8.
Diseases. 2015 Jun 19;3(2):111-121
Clin Diabetes Endocrinol. 2018 Dec 19;4:24.
Exp Ther Med. 2014 Dec;8(6):1906-1908. Epub 2014 Oct 15.
Pak J Med Sci. 2014 Mar;30(2):287-90.
Case Rep Med. 2012;2012:813461.
Prz Gastroenterol. 2018;13(3):249-250
J Clin Gastroenterol. 2019 Nov/Dec;53(10):e416-e423.
Clin Exp Gastroenterol. 2014 Dec 29;8:43-8
Eat Weight Disord. 2018 Oct 27.
Case Rep Pediatr. 2012;2012:269689
Middle East J Dig Dis. 2011 Mar;3(1):5-12.
Front Neurosci. 2017 Mar 27;11:155.
Case Rep Endocrinol. 2017;2017:2652403.
Ont Health Technol Assess Ser. 2011;11(3):1-63. Epub 2011 Jul 1.
J Community Hosp Intern Med Perspect. 2019 Feb 11;9(1):22-24
Mayo Clin Proc. 2016 Dec 5. pii: S0025-6196(16)30634-6.
Clin Transl Gastroenterol. 2019 May 22;10(5):1-10.
United European Gastroenterol J. 2018 Aug;6(7):1022-1031
Ann Hepatol. 2016 Jul–Aug;15(4):588-591.
Indian J Gastroenterol. 2019 Jun;38(3):263-267.
Nutrients. 2019 Aug 16;11(8). pii: E1925.
Indian J Med Res. 2019 Jan;149(1):18-25.
Hum Mol Genet. 2019 Sep 15;28(18):3037-3042
Acta Paediatr. 2019 Apr;108(4):676-680
J Clin Gastroenterol. 2019 Oct 31.
J Pediatr. 2016 Dec;179:131-138.e1
BMC Gastroenterol. 2015 Dec 21;15:181
Sci Rep. 2017 Mar 24;7:45286
Can J Diet Pract Res. 2018 Sep 1;79(3):118-124.
Pediatr Diabetes. 2012 Mar;13(2):163-9
Dig Dis Sci. 2012 May;57(5):1314-20.
Pediatr Diabetes. 2011 Jun;12(4 Pt 1):322-5
Indian J Med Res. 2019 Jan;149(1):18-25.
Nutr Diabetes. 2017 Jan 9;7(1):e239
Ann Hepatol. 2016 Jul-Aug;15(4):588-91
BMJ Open. 2015 May 11;5(5):e008097.
Vnitr Lek. 2014 Jul-Aug;60(7-8):562-6
J Paediatr Child Health. 2014 Oct;50(10):811-6.
BMC Gastroenterol. 2014 May 28;14:99.
Nutrients. 2013 Nov 27;5(12):4869-79.
Eur J Clin Invest. 2014 Jan;44(1):74-82
Pediatr Diabetes. 2013 Sep;14(6):455-8
Diabetes Educ. 2013 Jul-Aug;39(4):532-40
Diabet Med. 2013 Jul;30(7):835-9
Pediatr Diabetes. 2012 Dec;13(8):597-606
Acta Diabetol. 2013 Jun;50(3):319-24
Diabetes Care. 2011 Oct;34(10):2158-63
Pediatr Diabetes. 2011 Jun;12(4 Pt 1):322-5
Atherosclerosis. 2011 Aug;217(2):531-5.
J Pediatr Endocrinol Metab. 2010 Nov;23(11):1169-73.
Acta Biomed. 2010 Dec;81(3):165-70.
Tunis Med. 2010 Jan;88(1):18-22.
Przegl Lek. 2009;66(4):170-5.
Diabetologia. 2009 May;52(5):798-800
Bone. 2008 Aug;43(2):322-6
Diabetes Care. 2002 Jul;25(7):1117-22.

*Strategy #4*

- Search Query:
  *((“Celiac Disease”[Mesh] AND “Diabetes Mellitus, Type 1”[Mesh]) AND “Diet, Gluten-Free”[Mesh]) AND ((((“Glycated Hemoglobin A”[Mesh] OR “Bone Density”[Mesh]) OR “Quality of Life”[Mesh]) OR “Growth”[Mesh]) OR “Anemia, Iron-Deficiency”[Mesh])*

- Time of Search:
  *February 8, 2020. 11:54 AM.*

- Results:

*18.*

Nutr Diabetes. 2017 Jan 9;7(1):e239
J Pediatr. 2016 Dec;179:131-138
BMC Gastroenterol. 2015 Dec 21;15:181
BMJ Open. 2015 May 11;5(5):e008097
Diabetes Care. 2015 May;38(5):801-7
BMC Gastroenterol. 2014 May 28;14:99
Diabet Med. 2014 Feb;31(2):208-12
Diabet Med. 2013 Jul;30(7):835-9
Pediatr Diabetes. 2012 Dec;13(8):597-606
Acta Diabetol. 2013 Jun;50(3):319-24
Dig Dis Sci. 2012 May;57(5):1314-20
Pediatr Diabetes. 2012 Mar;13(2):163-9
Atherosclerosis. 2011 Aug;217(2):531-5
J Pediatr Endocrinol Metab. 2010 Nov;23(11):1169-73
J Gastroenterol Hepatol. 2011 Feb;26(2):378-81
Acta Biomed. 2010 Dec;81(3):165-70
Tunis Med. 2010 Jan;88(1):18-22
Diabet Med. 2009 Dec;26(12):1250-4

*Strategy #5*

- Search Query:
  *(“Celiac” OR “Coeliac” OR “Gluten Sensitive Enteropathy”) AND (“T1DM” OR “Type 1 DM” OR “Type 1 Diabetes Mellitus” OR “Juvenile Diabetes” OR “Insulin Dependent Diabetes”) AND (“GFD” OR “Gluten Free”) AND (“Glycemic Control” OR “HbA1C” OR “BMD” OR “Bone Mineral Density” OR “Bone Density” OR “QOL” OR “Quality of Life” OR “Growth” OR “Iron Deficiency Anemia”) NOT Medline[Sb]*

- Time of Search:
  *February 9, 2020. 6:59 PM.*

- Results:

*3.*

Diabetol Metab Syndr. 2016 Jul 29;8:51
World J Diabetes. 2015 Jun 10;6(5):707-14
World J Diabetes. 2013 Aug 15;4(4):130-4

D. Web of Science:

*Strategy #1*

- Search Query:
  *TS=(“type 1 diabetes mellitus” OR “dm type 1” OR “dmt1” OR “juvenile diabetes” OR “insulin-dependent diabetes mellitus” OR “diabetes mellitus type 1” OR “type 1 diabetes”) AND TS=(“gluten-free diet” OR “gluten free” OR “gfd” OR “special diet” OR “gluten free diet” OR “gluten-free”) AND ALL=(glycemic control OR blood glucose OR hba1c OR glucose tolerance test OR quality of life)*
  *Timespan: 2011-2019.*
  *Indexes: SCI-EXPANDED, SSCI, A&HCI, CPCI-S, CPCI-SSH, BKCI-S, BKCI-SSH, ESCI.*

- Time of Search:
  *October 18, 2019.*

- Results:

53.

International Journal of Endocrinology Volume 2019, 1-9
Pediatric Diabetes. 2019;1–10
European Journal of Inflammation,2019, Volume 17: 1–5
Journal of Functional Foods Volume 56, May 2019, Pages 163-170
PAEDIATRICS AND INTERNATIONAL CHILD HEALTH 2019, VOL. 39, NO. 1, 23–31
John Wiley & Sons Ltd. 2018 107, pp. 1879–1887
Velasco-Benítez CA/et al/Colombia Médica - Vol. 49 Nº4 2018 (Oct-Dec), 273-279
Pediatric Diabetes October 2018; 19 (Suppl. 27); 275-286
Diabetes Research and Clinical Practice Volume 143, September 2018, Pages 282-287
United European Gastroenterology Journal 2018, Vol. 6(7) 1022–1031
Journal of Child Psychology and Psychiatry, 60, 7, (803-812), (2018)
J Endocrinol Metab. 2018;8(2-3):37-42
The Lancet Child & Adolescent Health Volume 2, Issue 2, February 2018, Pages 133-143
Dig Dis 2018;36:399–408
British Journal of Hospital Medicine,2017, Vol. 78, No. 10
Acta Diabetologica October 2017, Volume 54, Issue 10, pp 889–894
ANNALS OF KING EDWARD MEDICAL UNIVERSITY LAHORE PAKISTAN, 2017, Volume: 23 Issue: 3 Pages: 307-311
Scientific Reports | 7:45286, 2017, 1-7
Adv Nutr 2017;8:356–61
JPGN Volume 64, Number 2, February 2017, 175-179
Nutrition & Diabetes (2017) 7, e239, 1-6
The Journal of Pediatrics Volume 179, December 2016, Pages 131-138
Indian Journal of Gastroenterology September 2016, Volume 35, Issue 5, pp 372–378
Diabetol Metab Syndr (2016) 8:51
SpringerPlus (2016) 5:994
ANNALS OF HEPATOLOGY,2016, Volume: 15 Issue: 4 Pages: 588-591
Gastroenterology (2015) 15:181
Canadian Journal of Diabetes Volume 39, Issue 6, December 2015, Pages 513-519
Nature Reviews Gastroenterology & Hepatology volume 12, pages 580–591 (2015)
Nutrients 2015, 7, 8733–8751
Nature Reviews Gastroenterology & Hepatology volume 12, pages 507–515 (2015)
WORLD JOURNAL OF DIABETES,2015, Volume: 6 Issue: 5 Pages: 707-714
Diabetes Care 2015;38:760–766
United European Gastroenterology Journal 2015, Vol. 3(2) 106–120
Journal of Pediatric Nursing Volume 30, Issue 2, March–April 2015, Pages 353-363
BMJ Open 2015;5:e008097, 1-8
Diabetes, Obesity and Metabolism 17: 3–8, 2015
Scandinavian Journal of Gastroenterology. 2014; 49: 1304–1310
J Paediatr Child Health,2014, 50: 811-816
Pediatr Diabetes,2014, 15: 270-278
BMC Gastroenterology 2014, 14:99
PLOS ONE, November 2013 | Volume 8 | Issue 11, 1-9
Acta Diabetologica October 2013, Volume 50, Issue 5, pp 821–822
Acta Diabetologica June 2013, Volume 50, Issue 3, pp 319–324
Der Diabetologe March 2013, Volume 9, Issue 2, pp 128–134
Pediatric Diabetes,2012, Volume 13, Issue 8, 597-606
Italian Journal of Pediatrics 2012, 38:10
Pediatric Diabetes,2012, 13: 163-169
European Journal of Gastroenterology & Hepatology: December 2011 - Volume 23 - Issue 12 - p 1270–1272
Diabetes Care 34:2158–2163, 2011
Atherosclerosis Volume 217, Issue 2, August 2011, Pages 531-535
Pediatric Diabetes,2011, 12: 322-325
J Pediatr. 2011 February; 158(2): 276–281

*Strategy #2*

- Search Query:
  *#1 (TS=(Celiac OR Coeliac OR Gluten Sensitive Enteropathy)) AND **LANGUAGE:** (English) AND **DOCUMENT TYPES:** (Article)*
  *Indexes=SCI-EXPANDED, SSCI, A&HCI, CPCI-S, CPCI-SSH, BKCI-S, BKCI-SSH, ESCI*
  *Timespan=All years*
  *#2 (TS=(T1DM OR Type 1 DM OR Type 1 Diabetes Mellitus OR Juvenile Diabetes OR Insulin Dependent Diabetes)) AND **LANGUAGE:** (English) AND **DOCUMENT TYPES:** (Article)*
  *Indexes=SCI-EXPANDED, SSCI, A&HCI, CPCI-S, CPCI-SSH, BKCI-S, BKCI-SSH, ESCI*
  *Timespan=All years*
  *#3 (TS=(GFD OR Gluten Free)) AND **LANGUAGE:** (English) AND **DOCUMENT TYPES:** (Article)*
  *Indexes=SCI-EXPANDED, SSCI, A&HCI, CPCI-S, CPCI-SSH, BKCI-S, BKCI-SSH, ESCI*
  *Timespan=All years*
  *#4 (TS=(Glycemic Control OR HbA1C OR BMD OR Bone Mineral Density OR Bone Density OR QOL OR Quality of Life OR Growth OR Iron Deficiency Anemia)) AND **LANGUAGE:** (English) AND **DOCUMENT TYPES:** (Article)*
  *Indexes=SCI-EXPANDED, SSCI, A&HCI, CPCI-S, CPCI-SSH, BKCI-S, BKCI-SSH, ESCI*
  *Timespan=All years*
  *#5 #4 AND #3 AND #2 AND #1*
  *Indexes=SCI-EXPANDED, SSCI, A&HCI, CPCI-S, CPCI-SSH, BKCI-S, BKCI-SSH, ESCI*
  *Timespan=All years*

- Time of Search:
  *January 31, 2020. 4:38 PM.*

- Results:

*80.*

PEDIATRICS DEC 2005;116(6):E754-E759
DIABETES CARE NOV 2006;29(11):2452-2456
CURRENT OPINION IN GASTROENTEROLOGY MAR 2010;26(2):116-122
AMERICAN JOURNAL OF CLINICAL NUTRITION MAR 1998;67(3):477-481
JOURNAL OF PEDIATRIC GASTROENTEROLOGY AND NUTRITION OCT 2001;33(4):462-465
CANADIAN JOURNAL OF GASTROENTEROLOGY AND HEPATOLOGY MAY 2001;15(5):297-301
DIABETES CARE JUL 2002;25(7):1117-1122
DIABETES CARE OCT 2011;34(10):2158-2163
ARCHIVES OF DISEASE IN CHILDHOOD SEP 2004;89(9):871-876
GASTROENTEROLOGY APR 2005;128(4)(suppl. 1):S52-S56
JOURNAL OF PEDIATRIC ENDOCRINOLOGY & METABOLISM MAY-JUN 1999;12(3):433-442
HEPATO-GASTROENTEROLOGY MAR-APR 2001;48(38):462-464
DIABETIC MEDICINE AUG 2005;22(8):1079-1082
POSTGRADUATE MEDICAL JOURNAL FEB 2007;83(976):132-136
ALIMENTARY PHARMACOLOGY & THERAPEUTICS APR 15 2009;29(8):898-905
JOURNAL OF PEDIATRIC GASTROENTEROLOGY AND NUTRITION SEP 2005;41(3):317-321
ACTA PAEDIATRICA 2002;91(3):297-302
JOURNAL OF PEDIATRICS MAY 2007;150(5):461-466
JOURNAL OF GASTROENTEROLOGY AND HEPATOLOGY FEB 2011;26(2):378-381
PEDIATRIC DIABETES JUN 2011;12(4pt1):322-325
ATHEROSCLEROSIS AUG 2011;217(2):531-535
PEDIATRIC DIABETES JUN 2007;8(3):171-176
WORLD JOURNAL OF GASTROENTEROLOGY AUG 28 2012;18(32):4399-4403
JOURNAL OF PEDIATRICS FEB 2011;158(2):276-U141
DIABETES CARE MAY 2015;38(5):760-766
PEDIATRICS SEP 2009;124(3):E489-E495
PEDIATRIC DIABETES DEC 2012;13(8):597-606
JOURNAL OF PEDIATRICS APR 2011;158(4):589-U94
DIABETIC MEDICINE DEC 2009;26(12):1250-1254
JOURNAL OF PEDIATRIC HEMATOLOGY ONCOLOGY FEB 2003;25(2):169-172
JOURNAL OF CLINICAL GASTROENTEROLOGY MAY-JUN 2008;42(5):460-465
CLINICAL AND EXPERIMENTAL RHEUMATOLOGY JAN-FEB 2012;30(1):126-131
DIABETOLOGIA JUL 2014;57(7):1339-1345
PEDIATRIC DIABETES AUG 2008;9(4pt1):277-284
DIABETIC MEDICINE JUL 2005;22(7):889-892
ACTA DIABETOLOGICA JUN 2013;50(3):319-324
HORMONE AND METABOLIC RESEARCH APR 2002;34(4):192-195
BEST PRACTICE & RESEARCH CLINICAL GASTROENTEROLOGY JUN 2005;19(3):479-486
PEDIATRIC DIABETES MAR 2012;13(2):163-169
SCANDINAVIAN JOURNAL OF GASTROENTEROLOGY MAR 3 2016;51(3):288-294
PEDIATRICS INTERNATIONAL FEB 2015;57(1):107-112
BMJ Open. 2015 May 11;5(5):e008097
JOURNAL OF PEDIATRIC GASTROENTEROLOGY AND NUTRITION FEB 2017;64(2):175-179
DIABETES EDUCATOR JUL 2013;39(4):532-540
JOURNAL OF PEDIATRICS 2016 Dec;179:131-138
PEDIATRICS INTERNATIONAL AUG 2010;52(4):579-583
BONE AUG 2008;43(2):322-326
JOURNAL OF PEDIATRIC GASTROENTEROLOGY AND NUTRITION SEP 2015;61(3):297-302
JOURNAL OF PEDIATRIC ENDOCRINOLOGY & METABOLISM NOV-DEC 2000;13(9):1629-1631
Bratisl Lek Listy. 2009;110(4):258-62
SAUDI MEDICAL JOURNAL DEC 2002;23(12):1514-1517
BMC Gastroenterol. 2014 May 28;14:99
ACTA DIABETOLOGICA OCT 2017;54(10):889-894
ACTA DIABETOLOGICA DEC 2015;52(6):1167-1174
JOURNAL OF PAEDIATRICS AND CHILD HEALTH OCT 2014;50(10):811-816
EUROPEAN JOURNAL OF GASTROENTEROLOGY & HEPATOLOGY NOV 2011;23(12):1270-1272
DIABETIC MEDICINE DEC 2014;31(12):E33-E36
JOURNAL OF PEDIATRIC ENDOCRINOLOGY & METABOLISM NOV 2010;23(11):1169-1173
ACTA PAEDIATRICA APR 2017;106(4):639-646
JOURNAL OF PEDIATRIC NURSING-NURSING CARE OF CHILDREN & FAMILIES MAR-APR 2015;30(2):353-363
PEDIATRIC DIABETES JUN 2018;19(4):741-748
PEDIATRIC DIABETES JUN 2018;19(4):749-755
Nutr Diabetes. 2017 Jan 9;7(1):e239
CANADIAN JOURNAL OF DIABETES SEP 2011;35(4):334-339
DIABETES RESEARCH AND CLINICAL PRACTICE SEP 2018;143():282-287
JOURNAL OF ENDOCRINOLOGY AND METABOLISM MAY 2018;8(2-3):37-42
INDIAN JOURNAL OF GASTROENTEROLOGY SEP 2016;35(5):372-378
Book (Capter 10:Extraintestinal Manifestations of Celiac Disease and Associated Disorders) https://doi.org/10.3926/oms.258
Book (Chapter: Celiac Disease in Adults) https://doi.org/10.3926/oms.233
Int J Endocrinol. 2019 Sep 19;2019:7895207
PEDIATRIC DIABETES DEC 2019;20(8):1100-1109
EUROPEAN JOURNAL OF INFLAMMATION JUN 2019;17():-
JOURNAL OF PEDIATRIC ENDOCRINOLOGY & METABOLISM JAN 2019;32(1):89-93
COLOMBIA MEDICA OCT-DEC 2018;49(4):273-279
PEDIATRIC GASTROENTEROLOGY HEPATOLOGY & NUTRITION DEC 2017;20(4):222-226
ANNALS OF KING EDWARD MEDICAL UNIVERSITY LAHORE PAKISTAN JUL-SEP 2017;23(3):307-311
GAZZETTA MEDICA ITALIANA ARCHIVIO PER LE SCIENZE MEDICHE MAY 2017;176(5):308-313
Book (Chapter 9.3: Coeliac Disease and diabetes) https://doi.org/10.1002/9781119121725.ch32
Book (Chapter:Extraintestinal Manifestations and Associated Diseases) https://doi.org/10.3926/oms.215
Przegląd Gastroenterologiczny 2011; 6 (4): 209–212

2. Other Databases

A. Google Scholar:

*Strategy #1*

- Search Query:
  ***with all of the words:***
  *“diabetes mellitus type 1” “gluten free” “glycemic” “glucose” celiac children*
  ***with the exact phrase:***
  *gluten-free diet*
  ***with at least one of the words:***
  ***without the words:***
  ***where my words occur:***
  ***Return articles authored by:***
  ***Return articles published in:***
  ***Return articles dated between:***
  *2011–2020*

- Time of Search:
  *October 18, 2019.*

- Results:

40

Nutrients 2015, 7, 7143-7162
Pediatr Diabetes. 2018 October; 19(Suppl 27): 275–286
Pediatr Diabetes. 2015 November; 16(7): 485–492
Ann. Pak. Inst. Med. Sci. 2018, 47-51
Human and Veterinary Medicine; Cluj-Napoca Vol. 9, Iss. 1, (Mar 2017): 11-15
Clinical Biochemistry 54 (2018) 11–17
European Journal of Endocrinology (2016) 174, R127–R138
Nat. Rev. Gastroenterol. Hepatol. 8, 405–415 (2011)
Google E-book Autoimmune associated diseases in pediatric patients with type 1 diabetes mellitus according to hla-dq genetic polymorphism
Oman Medical Journal (2015), Vol. 30, No. 2: 83–89
Autoimmunity Reviews 16 (2017) 712–721
The Impact of Coexisting Coeliac Disease on Type 1 Diabetes By Anna Pham-Short
American Society for Nutrition. Adv. Nutr. 4: 277–286, 2013
Diabetes Research and Clinical Practice Volume 123, January 2017, Pages 63-74
book Diabetic bone disease: Basic and translational research and clinical applications (pp.3-24)
Bakker-van Waarde, W. M. (2006). Bile salt and cholesterol metabolism in diabetes mellitus type 1
Google E-book Medical Management of Type 1 Diabetes Sixth Edition American Diabetes Association
Ontario Health Technology Assessment Series 2011; Vol. 11, No. 3, 1-63
Children 2018, 5, 169, 1-13
Book STUDY ON THE GLYCAEMIC CONTROL AND RELATED COMPLICATIONS IN TYPE 1 DIABETIC CHILDREN By Dr. HEMA G. R. MBBS
Google E-book Glucose Regulation, an issue of nursing clinics by Celia M. Levesque
Physiol. Res. 67 (Suppl. 3): S441-S454, 2018
Current Diabetes Reports October 2017, 17:83
Journal of Pediatric Endocrinology and Metabolism Volume 20, Issue 12
Feingold KR, Anawalt B, Boyce A et al., editors. Endotext [Internet]. South Dartmouth (MA): MDText.com, Inc.; 2000-
Feingold KR, Anawalt B, Boyce A et al., editors. Endotext [Internet]. South Dartmouth (MA): MDText.com, Inc.; 2000-
The Diabetes Textbook,2019, pp 941-966
Hindawi Publishing Corporation Journal of Diabetes Research Volume 2015, 1-20
A Practical Approach to Adolescent Bone Health,2018, pp 179-218
Book Wheat Syndromes How Wheat, Gluten and ATI Cause Inflammation, IBS and Autoimmune Diseases
Journal of Pediatric Endocrinology and Metabolism Volume 22, Issue 12
Critical Reviews™ in Immunology Volume 33, 2013 Issue 3, 245-281
Book MUDr. Michal Huml Dizertační práce: Vliv Gastrointestinálního traktu na kompenzaci diabetes mellitus typu 1 v dětském věku Gastrointestinální hormony
Diabetol Metab Syndr 2018, 10(Suppl 1):27
Current Opinion in Pediatrics: August 2010 - Volume 22 - Issue 4 - p 545–558
Zsu, Hma, Pzheng - pdfs.semantischolar.org (Link not found)
Book MA Weinberg, SL Segelnick, JS Insler, S Kramer -2015 Wiley online library (File not found)
Book Endocrinology A methodological approach towards integrative understanding BOLK’S COMPANIONS ON THE PRACTICE OF MEDICINE
Book COMPARATIVE ANALYSIS OF THE SOLUBLE WHEAT PROTEINS AND HUMAN HEALTH Nhu Tuyen Vu The University of Sydney March 2014
Book Analyza wybranych czynikow u dzieci z nowo rozpoznana cukryca typu 1 i ich wplyw na przebieg kliniczny choroby

*Strategy #2*

- Search Query:
  *
  with all of the words* *→* *celiac diabetes
  *
  *
  with the exact phrase* *→* *gluten free diet
  *
  *
  with at least one of the words* *→* *pediatrics children infants adolescent “diabetes type 1” “type 1 diabetes” “gluten free” diet regimen
  *
  *2011-2020*
  **Results listed below (42 references) include results of this search strategy plus papers retrieved through snowballing.*

- Time of Search:

*November 18, 2019.*

- Results:

*42.*

Diabetes Care 2011 Oct; 34(10): 2158-2163
World J Diabetes. Aug 15, 2013; 4(4): 130-134
Acta Diabetologica; Heidelberg Vol. 50, Iss. 5, (Oct 2013): 821-2.
*Nutrients 2015, 7(9), 7143-7162*
J Pediatr. 2011 February; 158(2): 276–281
The Journal of Pediatrics. April 2011, 158, (4):Pages 589-593.e2
Pediatr Diabetes. 2011 Jun;12(4 Pt 1):322-5
Pediatr Diabetes. 2012 Dec;13(8):597-606.
Diabetes Care 2015 May; 38(5): 760-766.
BMC Gastroenterology volume 14, Article number: 99 (2014)
BMC Gastroenterol. 2015 Dec 21;15:181
Diabetes Care 2016 Aug; 39(8): e119-e120
Acta Bio Med 2011, 81, 165-170
Journal of Pediatric Gastroenterology and Nutrition. 41(3):317-321, SEPTEMBER 2005
Pediatric Diabetes. 10():92–93, SEPTEMBER 2009
J ASEAN Fed Endocr Soc, 2016, 31(1): 5-9
Berioli, M. G., Mancini, G., Principi, N., Santi, E., Ascenzi, M., Rogari, F., … Esposito, S. (2019). Growth and glycemic control in children with type 1 diabetes and asymptomatic celiac disease treated with a gluten -free diet for 1 year. European Journal of Inflammation. https://doi.org/10.1177/2058739219855574
Gastroenterology Rev 2018; 13 (3): 249–250
AGA.May 2009,136, (5)(Suppl 1):A-473,
Diabetes Research and Clinical Practice,June 2009,84,(3), 332-334
*The Journal of Clinical Endocrinology & Metabolism, Volume 88, Issue 1, 1 January 2003, Pages 162–165,*
Gastroenterol Clin Biol. 2010 Apr-May;34(4-5):319-20
Clin Nutr. 2004 Apr;23(2):281-2.
La Pediatria Medica e Chirurgica: Medical and Surgical Pediatrics, 01 Mar 2007, 29(2):99-104
*Pediatric Diabetes, 2(3), 95–97*
Journal of Pediatric Endocrinology & Metabolism, 9, 101-111 (1996)
pediatric diabetes Volume12, Issue4pt1 June 2011
Clin Pediatr Endocrinol 1998; 7(2), 125-129
Hormone Research. February 2002; 57,(Suppl. 1):97-100
Diabetic Medicne.December 2009, 26;(12): 1250-1254
Diabetes Care 1998 Aug; 21(8): 1379-1380.
Diabetes Care 2006 Nov; 29(11): 2452-2456.
Diabetic medicine.1998;15,(1):38-44
Diabetic Medicine. 2005;22(8),1079-1082
acta paediatrica. March 2002;91(3):297-302
Journal of Pediatric Endocrinology & Metabolism, 12,433-442 (1999)
World J Diabetes. 2013 Aug 15; 4(4): 130–134.
Acta Biomed. 2010 Dec;81(3):165-70
Acta Diabetol 2013;50:319–324
BMC Gastroenterology,December 2014, 14:99
Prz Gastroenterol. 2018;13(3):249-250
J Pediatr Endocrinol Metab. 2010 Nov;23(11):1169-73

B. Springer:

- Search Query:
  *(diabetes mellitus type 1 OR juvenile diabetes OR dmt1 OR dm-1 OR insulin dependent diabetes) AND (Celiac OR coeliac OR gluten sensitive OR gluten enteropathy) AND (gluten free OR gfd OR special diet) AND (glycemic control OR hba1c OR blood glucose OR glucose tolerance test OR blood glucose control)*
  *Limiters used: ‘Article Type’ ‘English Language’ ‘2011–2020′*

- Time of Search:
  *October 12, 2019.*

- Results:

*29.*

Diabetologia September 2019, Volume 62, Supplement 1, pp 1–600
Diabetologia (2014) 57:[Suppl1]S1–S566
Internal and Emergency Medicine December 2012, Volume 7, Supplement 4, pp 361–568
Journal of General Internal Medicine May 2019, Volume 34, Supplement 2, pp 99–867
Internal and Emergency Medicine December 2011, Volume 6, Supplement 2, pp 191–392
Journal of General Internal Medicine,2017, Vol. 32, Issue 2, S83-S808
European Journal of Pediatrics November 2016, Volume 175, Issue 11, pp 1393–1880
Journal of General Internal Medicine,2014, Vol. 29, Issue 1, S1- S545
Journal of General Internal Medicine, 2018, Vol. 33, Issue. 2, S1- S758
Journal of General Internal Medicine, 2015, Vol. 30, Issue. 2, S45-S551
Hepatology International June 2013, Volume 7, Supplement 1, pp 1–754
Journal of General Internal Medicine, 2016, Vol. 31, Issue. 2, S85-S922
Obesity Surgery August 2011, Volume 21, Issue 8, pp 956–1156
Journal of General Internal Medicine June 2013, Volume 28, Supplement 1, pp 1–489
Osteoporosis International April 2018, Volume 29, Supplement 1, pp 149–565
Indian Journal of Gastroenterology October 2018, Volume 37, Supplement 1, pp 1–137
Internal and Emergency Medicine December 2011, Volume 6, Supplement 2, pp 141–190
Inflammation Research June 2011, Volume 60, Supplement 1, pp 1–321
Hepatology International March 2015, Volume 9, Supplement 1, pp 1–391
Journal of Inherited Metabolic Disease September 2015, Volume 38, Supplement 1, pp 35–378
Journal of Neurology June 2013, Volume 260, Supplement 1, pp 1–280
Journal of Inherited Metabolic Disease September 2016, Volume 39, Supplement 1, pp 35–284
Internal and Emergency Medicine December 2012, Volume 7, Supplement 4, pp 309–359
Journal of Neurology June 2012, Volume 259, Supplement 1, pp 1–236
Indian Journal of Gastroenterology November 2013, Volume 32, Supplement 1, pp 1–132
Italian Journal of Pediatrics 2017, 43(Suppl 2):110, 1-42
Journal of Inherited Metabolic Disease September 2018, Volume 41, Supplement 1, pp 37–219
Irish Journal of Medical Science (1971 -) March 2018, Volume 187, Supplement 3, pp 17–113
Advances in Rheumatology 2018, 58(Suppl 1):23, 1-115

3. Snowballing

- Results:

*2.*

J Pediatr Gastroenterol Nutr. 2001 Jan;32(1):37-40.
Endocrine Abstracts (2011);27:58

**Table A1:** Table of Quality Assessment GRADE.

Reference	Sun et al., 2009 [12]	Mackinder et al., 2014 [13]	Rami et al., 2005 [14]	Amin et al., 2002 [15]	Berioli et al., 2019 [17]	Saadah et al., 2004 [16]
Study Design	Observational	Observational	Observational	Observational	Observational	Observational
Risk of Bias	None Found	None Found	None found	Not serious	Serious	Serious
Inconsistency	None	None	None	Serious	Not serious	Not serious
Indirectness	None	None	None			
Imprecision	None	None	None	Serious	Serious	Serious
Publication Bias	None	None	None	None	None	None
Large Effect	No	No	No	No	No	No
Dose Response (Gradient)	Yes (A 5-year gradient is shown)	Yes (A 4-year gradient is shown)	Yes	Yes	Yes	Yes
All Plausible Confounding	-	-	-	-	-	-
Strength	Moderate (3)	Moderate (3)	Moderate (3)	Low (2)	Low (2)	Low (2)
